# Newcastle viral disease causation web correlations with laying hen productivity

**DOI:** 10.1038/s41598-024-65854-z

**Published:** 2024-07-11

**Authors:** Mohammed Abdelhameed Mohammed Kamal, Mohamed Atef, M. A. Khalf, Zakia A. M. Ahmed

**Affiliations:** Department of Veterinary Hygiene and Management, Faculty of Veterinary Medicine, Cairo University, Giza, 12211 Egypt

**Keywords:** Climate change, Egg production, Egg weight, Outbreaks, Poultry welfare, Sustainability, Climate sciences, Environmental sciences

## Abstract

Environmental conditions profoundly impact the health, welfare, and productivity of laying hens in commercial poultry farming. We investigated the association between microclimate variations, production indices, and histopathological responses to accidental Newcastle disease virus (NDV) infection within a controlled closed-house system. The study was conducted over seven months in a laying hen facility in Cairo, Egypt. Microclimate measurements included temperature, relative humidity (RH%), air velocity (AV), and the temperature humidity index (THI) that were obtained from specific locations on the front and back sides of the facility. Productivity indices, including the egg production percentage (EPP), egg weight (EW), average daily feed intake, and feed conversion ratio, were assessed monthly. During an NDV outbreak, humoral immune responses, gross pathology, and histopathological changes were evaluated. The results demonstrated significant (*p* < 0.05) variations in EPP and EW between the front and back sides except in April and May. AV had a significant (*p* = 0.006) positive effect (Beta = 0.346) on EW on the front side. On the back side, AV had a significant (*p* = 0.001) positive effect (Beta = 0.474) on EW, while it negatively influenced (*p* = 0.027) EPP (Beta = − 0.281). However, temperature, RH%, and THI had no impact and could not serve as predictors for EPP or EW on either farm side. The humoral immune response to NDV was consistent across microclimates, highlighting the resilience of hens. Histopathological examination revealed characteristic NDV-associated lesions, with no significant differences between the microclimates. This study underscores the significance of optimizing microclimate conditions to enhance laying performance by providing tailored environmental management strategies based on seasonal variations, ensuring consistent airflow, particularly near cooling pads and exhaust fans, and reinforcing the importance of biosecurity measures under field challenges with continuous monitoring and adjustment.

In poultry farming, the macro and microclimates within layer houses play a pivotal role in shaping the health, welfare, and productivity of laying hens. Microclimate conditions, including temperature, humidity, air quality, and airflow dynamics, directly influence the physiological processes and behaviors of hens, ultimately impacting their production performance and susceptibility to diseases^1^. Previous research has highlighted the intricate relationship between microenvironment variations and their effects on laying performance in commercial poultry operations^2^. However, few studies have specifically explored how microclimate dynamics interact with cage position within a controlled closed-house system and their combined impact on laying hen productivity and immune responses.

The microclimate within a closed-house system is influenced by various factors, including the distribution of airflow, temperature gradients, and humidity levels^3,4^. In particular, the arrangement of cages within the house can create microclimate variations, with differences observed between cage positions near cooling pads, exhaust fans, and inlet areas^5,6^. The presence of cold air near the cooling pad leads to continuous airflow toward the exhaust fan, allowing the cage to cool more effectively near the fan. Understanding these microclimate dynamics is essential for optimizing environmental conditions to support optimal laying performance. Layer hens, which are typically housed in intensive cage systems, are particularly sensitive to microclimatic changes, especially variations in temperature and humidity^7^. Climate change further complicates the situation, affecting poultry production both indirectly by influencing production inputs and directly by causing productivity losses^8^. Heat stress, a common challenge in poultry production, can lead to reduced feed intake, poor feed conversion efficiency, and decreased egg production^9–11^. Production indices, such as the ADFI, BW, EW, and EPP, are vital measures of productivity in the layer industry^12^. Moreover, fluctuations in microclimate conditions can impact the immune system of laying hens, affecting their resilience to infectious diseases such as Newcastle disease virus (NDV)^2,13,14^. Accidental outbreaks of NDV pose significant threats to poultry health and productivity, with histopathological changes observed in infected birds, including epithelial cell degeneration, inflammation, and organ damage^15^. The lungs often show edema, congestion, and hemorrhages, with alveolar rupture and inflammatory cell infiltration. Tracheal lesions include edema, desquamation of the lining epithelium, and inflammatory cell infiltration^16,17^. Understanding how microclimate variations influence the susceptibility and immune responses of laying hens to NDV infection is essential for developing effective disease management strategies^13^.

Thus we implemented this study to examine the complex interassociations between microclimate variations, cage position, and their effects on production performance and immune responses in laying hens within a controlled closed-house system. By elucidating these relationships, we aim to provide valuable insights for optimizing microclimate conditions and enhancing the welfare and productivity of laying hens in commercial poultry farming operations.

## Materials and methods

The Cairo University Institutional Animal Care and Use Committee (CU-IACUC), Veterinary Medical and Agricultural Sciences Sector granted ethical approval for this work under the code “VET CU 09092023767”. The “Guide for the Care and Use of Laboratory Animals”, issued by the Institute of Laboratory Animal Research, was followed by the Faculty of Veterinary Medicine at Cairo University for the procedures involving all the animals in this study (Washington, DC, USA). All animal procedures carried out in this study adhered to the ARRIVE 2.0 guidelines.

### Laying hens

In a dedicated commercial house for egg laying, 25,600 Hy-line W-80 white hens were used primarily for egg production. The study used birds at 280 days and 1.760 kg average BW. They started with a daily egg production of 23,550 eggs, with an average laying rate of 92% and an average egg weight of 64 g.

### Location

The study was conducted from November 2022 to May 2023 in a privately owned commercial laying hen facility in the eastern region of Cairo, Egypt. This facility is situated at approximately 30.17° N latitude and 31.61° E longitude, with an average altitude of approximately 132 m above sea level.

### The farm’s structural specifications

It has a height of 2.76 m with a ridge height of 2.9 m, spanning 85 m in length and 14 m in width, resulting in a floor area of 1190 m^2^.

### Cage system

**Figure 1:**
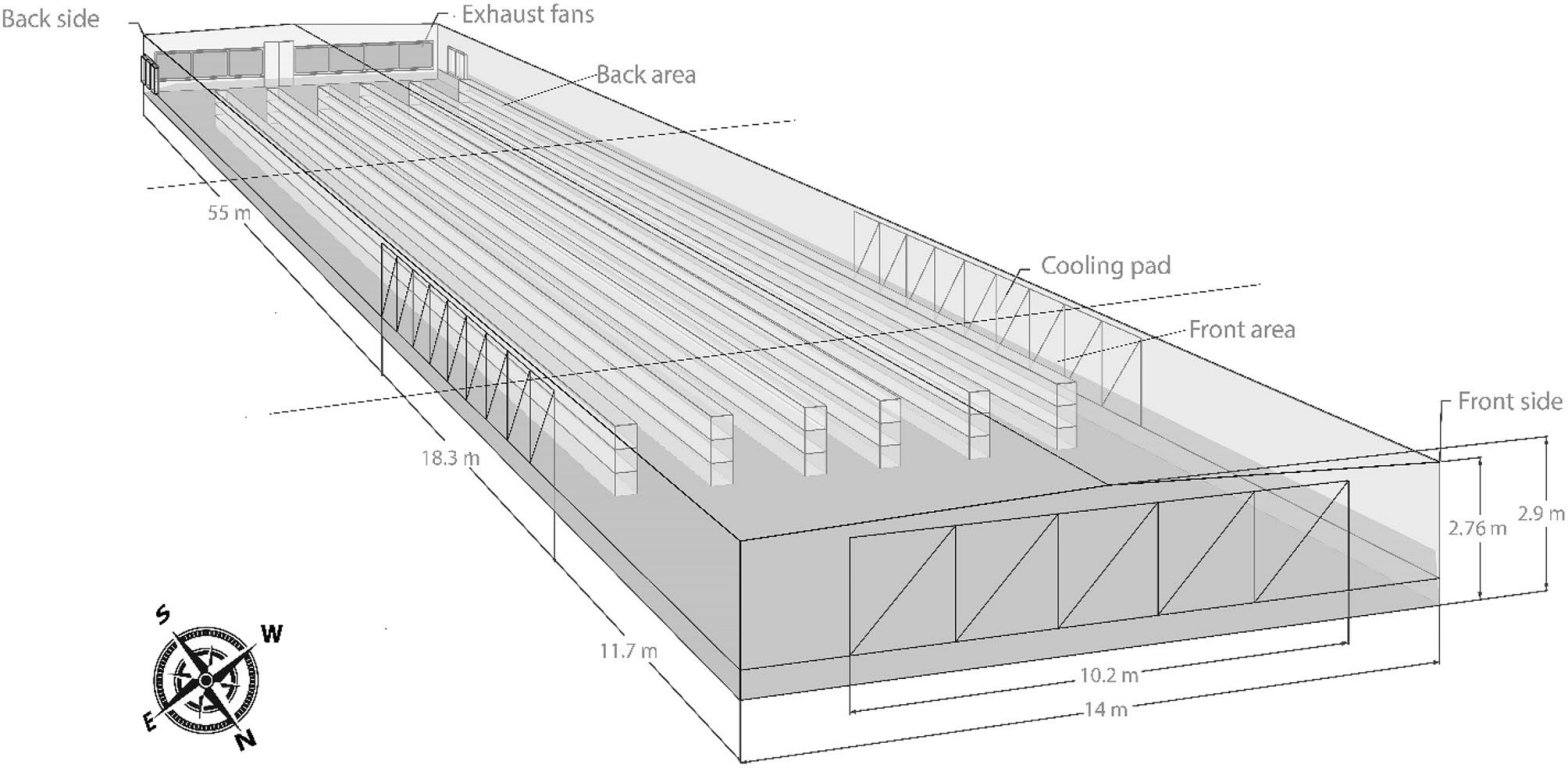
A schematic illustration of the chicken house’s construction.

Cage systems are widely used in commercial egg-laying operations due to their efficiency and egg-management capabilities. The cages were arranged in a layout of 6 rows and 3 tiers to make the most available space. Figure 1 provides a visual representation of the house's design. These three-tiered cage systems allow for the efficient use of vertical space in poultry houses. Each cage measured 60 cm in length, 50 cm in width, and 45 cm in height. Each cage housed 8 birds, and each tier had its egg collection belt and a feeding chain row situated in front of the cages, along with a dropping belt underneath them.

### Ventilation and cooling system

This study implemented a ventilation and cooling system that directed outdoor air through cellulose cooling pads with a total surface area of 20.4 m^2^ for effective climate control. These pads, located on the north wall and the front two sides of the house, facilitate airflow at 75 m/min. After passing through the cooling pads, the air traveled 85 m before being expelled by 13 extraction fans on the back side of the facility. Each fan had a substantial airflow rate of approximately 39,500 m^3^/h under a static pressure of 2.5 mm.

### Efficient management

Daily management procedures were conducted, including feeding, egg collection, and the evaluation of production indices (EPP and EW). Additionally, daily postmortem examinations were performed on morbid hens. Periodic activities included prophylactic drug administration, vaccination programs, and the provision of vitamins and minerals. Automatic feeding was carried out twice daily, following breed-specific instructions, while water was provided ad libitum from a commercial tap water source through adjustable nipple drinkers. A lighting system following the breed guidelines was implemented when the hens reached 40 weeks of age. This involved 16 h of light and 8 h of darkness, with a light intensity of 20–25 lx.

### Study design and microclimatic element measurements

Fixed cohorts (observational) closed populations of apparently healthy birds in which no additional birds were introduced during the observation periods were the subject of the design^18^. The study involved measuring and recording the air temperature (°C), RH%, and AV (m/s) at fourteen specific locations within the laying hen facility. These readings were collected from seven aisles positioned 14 m from the cooling pads on the front side of the farm and seven aisles positioned 14 m before the extracting fans on the back side of the farm.

A Lurton LM8100 microclimate measurement instrument (Lurton Electronic in Taiwan, temperature; 0 to 50 °C ± 0.1 °C, RH%; 10 to 95% ± 0.1%, and AV; 0.4 to 30.0 m/s ± 0.1 m/s), renowned for its accuracy, was used for these measurements. These measurements were collected daily at 11:00 AM over seven months. The THI was calculated using an equation modified by Marai et al.^19^. This equation takes into account the dry bulb temperature (db °C) and RH%.

### Evaluation of hen production performance indices

The study involved only two groups, 4424 birds on the front side, which were 6 replicates from the front of the 6 cage rows, and 4424 birds on the back side, which were 6 replicates from the back of the 6 cage rows of the farm, where all pairwise comparisons were made. This study monitored the productivity of laying hens, which included factors such as hen-day EPP, ADFI, and EW. The hen-day EPP was recorded by a separate collection of eggs from each row on the front and back sides, while the ADFI was recorded entirely on the front and back sides of each row. FCR was calculated by dividing the feed intake by egg mass (g/g) and was computed after Torki et al.^20^.

The study unit comprises multiple birds (a cage system), so the sample size was less than the number of birds on each side of the farm.

### Laboratory investigation

#### Humoral immune response evaluation

The HI test was used to assess the effectiveness of vaccinations and confirm outbreaks of NDV, H5, and H9. At 57 weeks of age, during a 3-week sudden accidental outbreak in March, blood samples were collected to evaluate the humoral immune response in hens. A total of one hundred and twenty blood samples were collected, with 60 collected from the front side and 60 from the back side of the facility. The sample size was calculated using online software (Experimental Design Assistant; https://eda.nc3rs.org.uk/eda/login/auth). The power of the experiment was set to 80%. A total of 50 birds per group were considered.

These samples were collected through puncture of the Vena ulnaris and promptly transferred to 2 ml microcentrifuge tubes (Carl Roth GmbH) and transported in an icebox. The blood samples were centrifuged at 12,000 rpm/3 min following Calnek^21^. The sera were carefully preserved at − 20 °C for future use. Subsequently, an HI test was conducted to assess the presence of hemagglutination-inhibiting antibodies against NDV and avian influenza H5 and H9 in the collected sera. Twofold serial dilutions of the sera were prepared and combined with an equal volume of the viral antigen following Oberlander et al.^22^. Chicken red blood cells were introduced into these dilutions, and the mixture was meticulously examined to determine if complete inhibition of hemagglutination was observed. According to Afonso et al.^23^, results falling below a logarithmic value of 4 are considered nonprotective.

In addition to the HI test, a commercial indirect ELISA kit (IDEXX Laboratories) was utilized to assess the samples and determine the titer of IBV antibodies following the manufacturer's instructions as per WOAH, 2009 guidelines^24^.

#### Postmortem and histopathological investigation

During the 3-week outbreak, the study focused on obtaining samples from both the front and back sides of the infected flock, each consisting of five birds. Following PM examination, samples were collected from the trachea, lung, and proventriculus, preserved in 10% neutral buffered formalin, dehydrated in alcohol, cleared in xylene, and embedded in paraffin blocks. The sections, approximately 5 µm thick, were obtained using a microtome. The sections were stained with the routine hematoxylin and eosin (H&E) following Bancroft et al.^25^.

#### Lesion scoring

A semiquantitative lesion scoring system was used to assess the infected tissues. For each of the five birds (n = 5), ten random optical fields were scrutinized and scored. These scores were then used to calculate the mean, along with the associated standard error (SEM), using SPSS software. In particular, the trachea was assessed on a scale ranging from zero to five, as described by Hussein et al., with the following criteria: zero represented normal tissue; 1 represented hyperemia and inflammatory cell infiltration; 2 represented hyperemia, inflammatory cell infiltration, and edema; 3 represented hyperemia, inflammatory cell infiltration, edema and deciliation; 4 represented slight hyperplasia and deciliation; and 5 represented hemorrhagic patches, desquamation and hyperplasia^26^. For lung evaluation, the scale ranged from 0 to 3, where 0 indicated normal lung tissue, 1 denoted inflammatory cell infiltration in the air capillaries, 2 indicated inflammatory cell infiltration, hemorrhage, and exudate in the secondary bronchi, and 3 represented hypertrophy of the tertiary bronchial epithelium and interstitial edema. The proventriculus was rated from 0 to 4, with 0 indicating normal tissue, 1 representing mild epithelial cell degeneration and necrosis with heterophils, 2 denoting extensive epithelial cell degeneration and necrosis accompanied by mononuclear cell infiltration, 3 signifying destruction of the lymphoid areas often associated with fibrin, and 4 indicating fully destroyed lymphoid areas with some hemorrhage.

#### Statistical analysis

For the data analysis process, Statistical Package for Social Sciences software, version 25.0 (SPSS Inc., Chicago, IL), was used. Initially, all collected information was encoded into variables for systematic analysis. The statistical analysis employed a fixed effect model to evaluate the effect of the indoor microclimatic parameters on hen-day EPP and EW. The model was specifically designed to test the effects of the microclimate on the front and back sides of the farm. The normality of the data was assessed through the Kolmogorov‒Smirnov test. Both descriptive and inferential statistics, including the independent sample t-test, Mann‒Whitney U test, ANOVA, Spearman's correlation, and linear regression, were employed to present and interpret the results. Additionally, a post hoc least significant difference (LSD) test was performed on the obtained results. For each statistical test, a significance level of less than 0.05 was considered to indicate statistical significance^27^.

## Results

### Production indices

**Figure 2:**
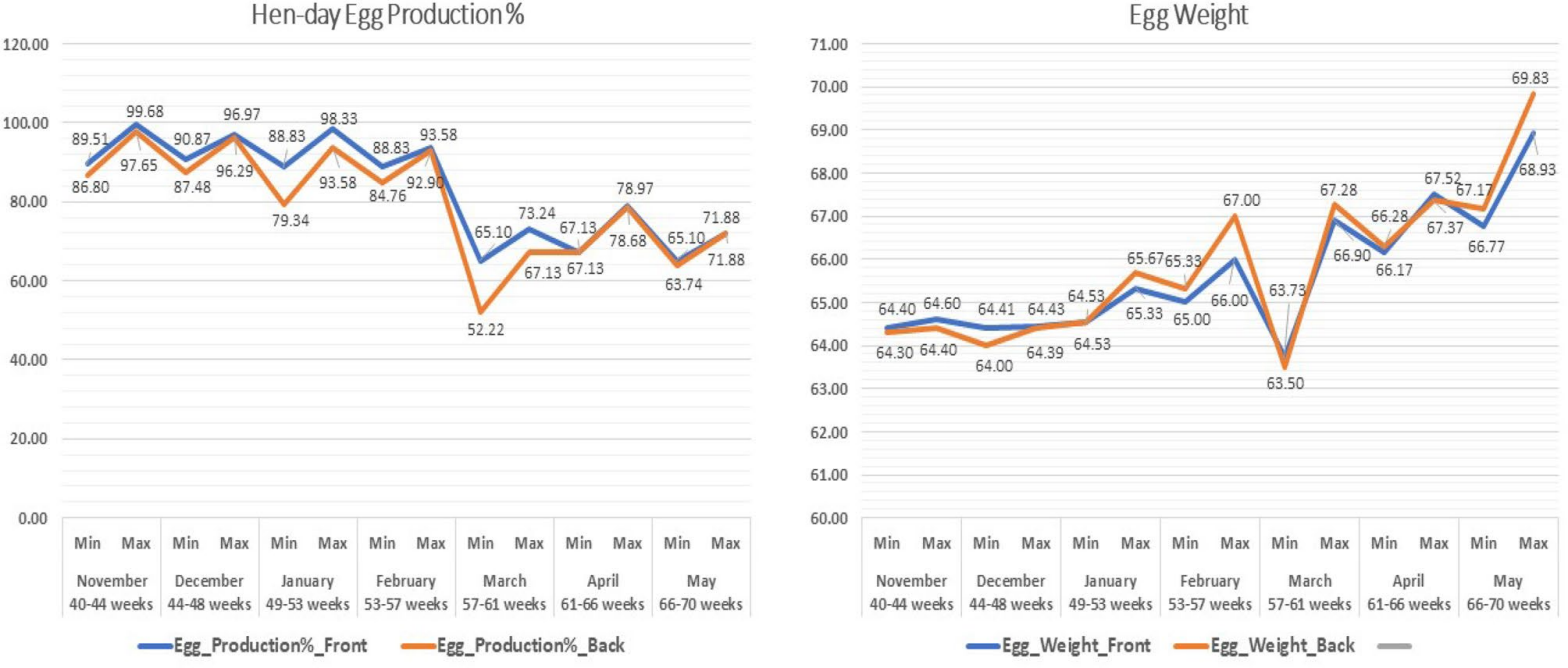
Maximum and minimum values of egg production % and egg weight during the study 7 months in the farm indoor front, and indoor back sides.

The study revealed significant (*p* < 0.05) variations in the production indices between the indoor front and back sides of the layer farm (Fig. 1), particularly for EPPs and EWs (Table 1 and Fig. 2). The results for each month were as follows:

**Table 1:** The mean ± SE of the hen-day egg production % and egg weight during the study (7 months) on the front and back sides of the layer farms and their statistical significance.

Parameters	Front side	Back side	Sig.
**November**			
Production %	94.36 ± 0.87	91.74 ± 0.95	0.052
Egg Weight (g)	64.49 ± 0.02	64.35 ± 0.01	0.000
Feed Intake (g/hen)	113.04 ± 0.16	113.04 ± 0.16	1.000
FCR	1.75 ± 0.00	1.76 ± 0.00	0.282
**December**			
Production %	95.37 ± 0.39	92.76 ± 0.56	0.001
Egg Weight (g)	64.42 ± 0.00	64.28 ± 0.05	0.006
Feed Intake (g/hen)	116.59 ± 0.17	116.59 ± 0.17	1.000
FCR	1.81 ± 0.00	1.81 ± 0.00	0.258
**January**			
Production %	93.14 ± 0.76	88.64 ± 1.15	0.003
Egg weight	64.84 ± 0.09	64.98 ± 0.11	0.339
Feed intake (g/hen)	116.91 ± 0.16	116.91 ± 0.16	1.000
FCR	1.80 ± 0.00	1.80 ± 0.00	0.455
**February**			
Production %	91.25 ± 0.37	88.88 ± 0.57	0.002
Egg weight (g)	65.48 ± 0.08	66.19 ± 0.12	0.000
Feed intake (g/hen)	116.64 ± 0.15	116.64 ± 0.15	1.000
FCR	1.78 ± 0.00	1.76 ± 0.00	0.000
**March**			
Production %	68.64 ± 0.80	59.05 ± 1.30	0.000
Egg weight	65.42 ± 0.27	65.22 ± 0.30	0.615
Feed intake (g/hen)	115.33 ± 0.11	115.33 ± 0.11	1.000
FCR	1.76 ± 0.01	1.77 ± 0.01	0.647
**April**			
Production %	73.62 ± 2.07	73.63 ± 1.92	1.000
Egg weight	66.97 ± 0.14	66.70 ± 0.10	0.131
Feed intake (g/hen)	113.49 ± 0.20	113.49 ± 0.20	1.000
FCR	1.69 ± 0.00	1.70 ± 0.00	0.11
**May**			
Production %	69.02 ± 0.43	68.44 ± 0.55	0.416
Egg weight	68.13 ± 0.20	68.45 ± 0.23	0.294
Feed intake (g/hen)	112.79 ± 0.09	112.79 ± 0.09	1.000
FCR	1.66 ± 0.01	1.65 ± 0.01	0.327

In November, the EPP was slightly greater on the front than on the back side, although the difference was not statistically significant. However, EW showed a significant (*p* < 0.001) difference, with the front side having slightly heavier eggs. In December, there was a notable difference in the EPP between the two sides, with the front significantly (*p* = 0.001) outperforming the back side. Similarly, the EW on the front side was significantly (*p* = 0.006) higher. January indicated a substantial gap in the EPP, with the front significantly (*p* = 0.003) surpassing the backside. EW, however, did not differ significantly between the two sides. February demonstrated substantial discrepancy in the EPP, with the front outperforming (*p* = 0.002) the backside. However, the EW on the front side was significantly (*p* < 0.001) lower.

March experienced a remarkable decrease in EPP on both sides. However, the front side retained a greater (*p* = 0.000) EPP. EW did not show a significant difference. In April and May, there were no significant differences in EPP or EW between the front and back sides. However, the ADFI (g/hen) remained constant between the front and back sides, and the FCR significantly differed (*p* < 0.001) between the front and back sides only during February.

### Climatic parameters as predictors

**Figure 3:**
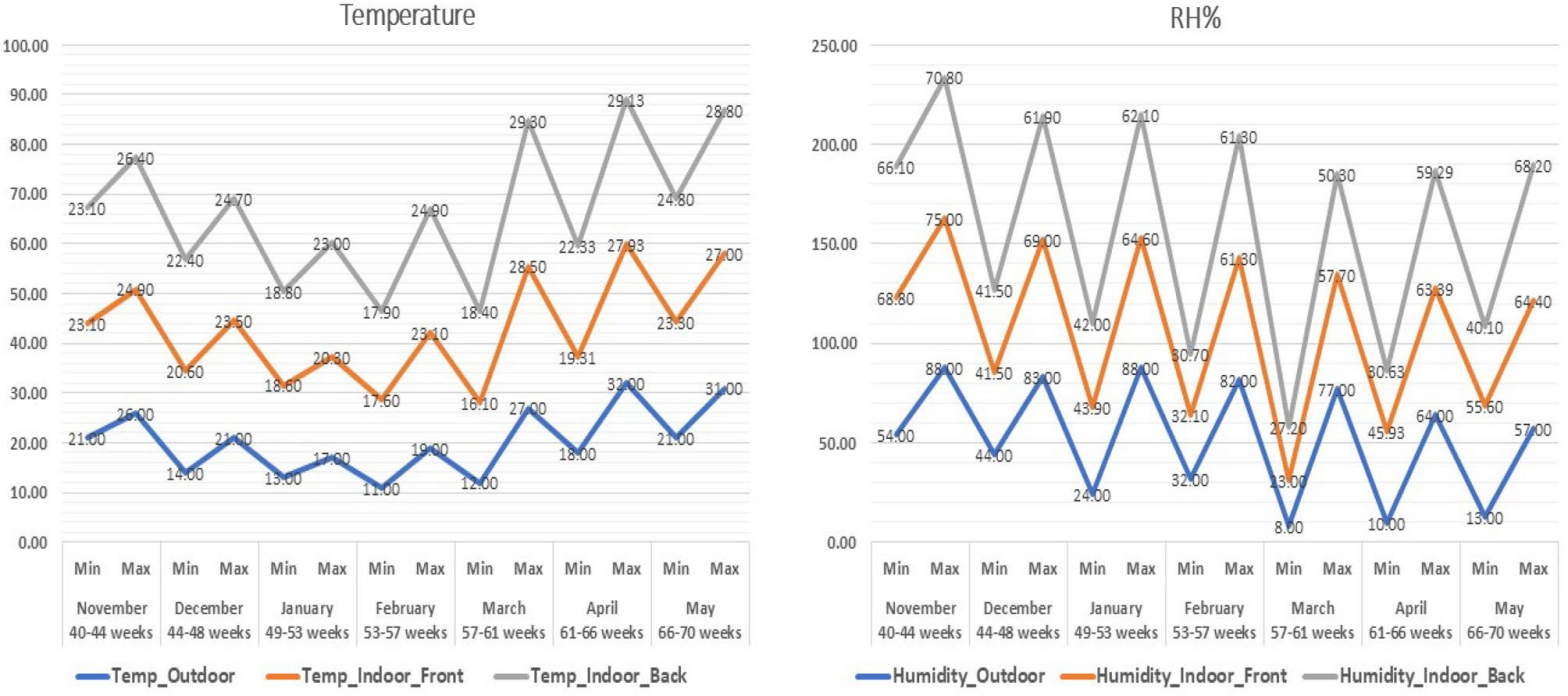
Maximum and minimum values of temperature (T°C) and RH% during the study 7 months in the farm outdoor climate, indoor front, and indoor back.

**Figure 4:**
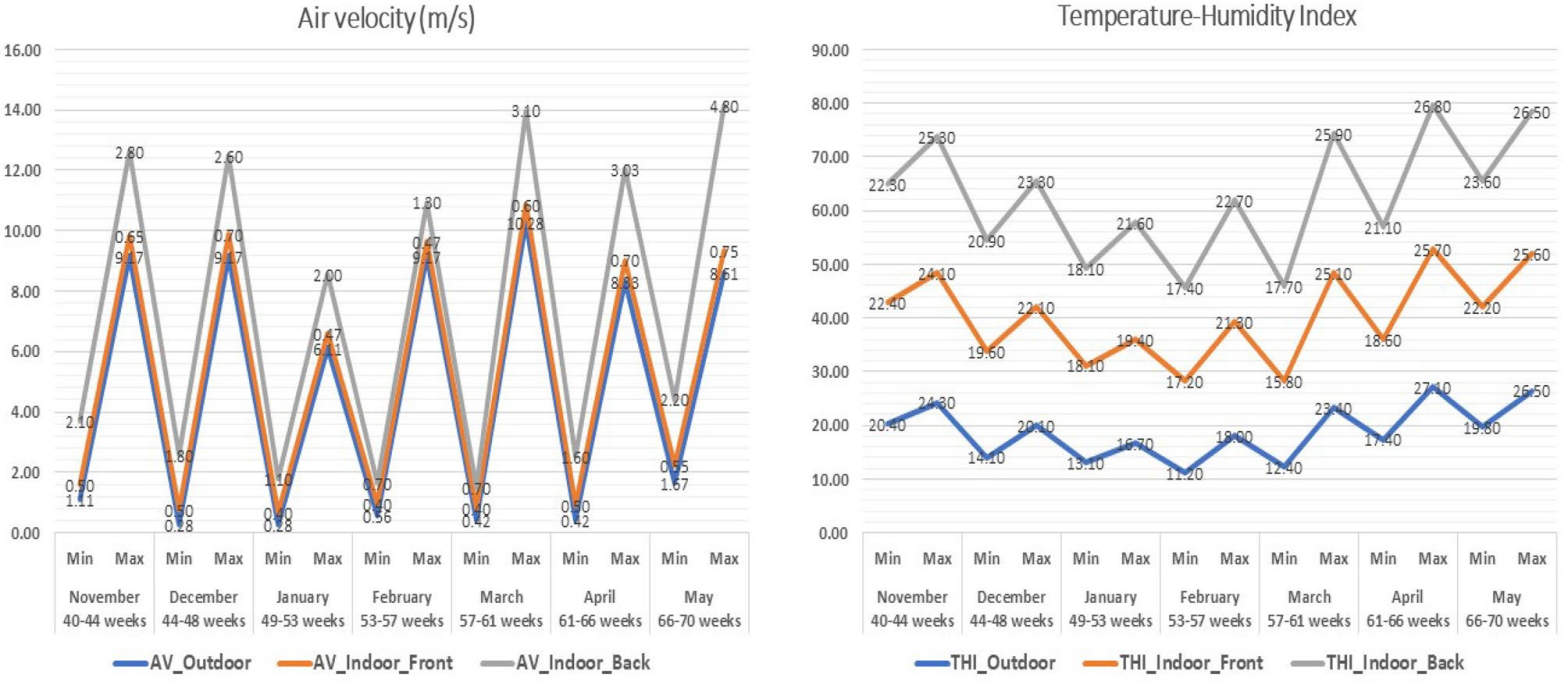
Maximum and minimum values of air velocity (m/s) and temperature-humidity index (THI) during the study 7 months in the farm outdoor climate, indoor front, and indoor back.

**Figure 5:**
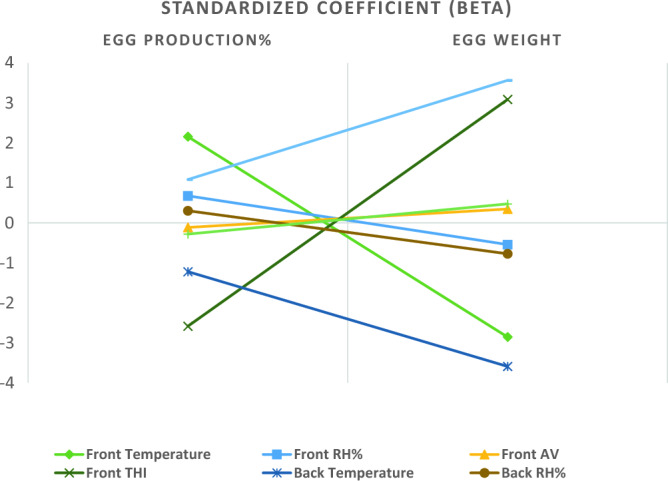
Standardized Coefficient (Beta) of indoor climatic predictors for hen-day egg production % and egg weight in the front and back sides of the layer farm.

The maximum and minimum temperature, RH%, AV, and THI values are shown in Figs. 3 and 4, emphasizing the variations in both the interior front and back sides in response to external climatic conditions. Linear regression analysis revealed that AV had a significant (*p* = 0.006) positive effect (Beta = 0.346) on EW on the front side (Table 2). On the back side (Table 2), AV had a significant (*p* = 0.001) positive effect (Beta = 0.474) on EW, while it negatively influenced (*p* = 0.027) EPP (Beta = − 0.281) (Fig. 5). Figure 5 shows the trends of the linear regression outputs that describe the indoor climatic factors as predictors for both the hen-day EPP and EW on the front and back sides of the farm.

**Table 2:** Standardized coefficient (Beta) values with their statistical significance of the farm front and back side climatic parameters as a predictor for the front and back side hen-day egg production % and egg weight.

Item		Egg production %	Sig	Egg weight	Sig.
T°C	Front side	2.158	0.371	− 2.854	0.250
RH%		0.676	0.060	− 0.544	0.139
AV		− 0.114	0.348	0.346	0.006
THI		− 2.586	0.299	3.088	0.228
T°C	Back side	− 1.221	0.650	− 3.591	0.226
RH%		0.303	0.519	− 0.774	0.135
AV		− 0.281	0.027	0.474	0.001
THI		1.085	0.695	3.565	0.241

### Immune responses and histopathological changes

During an NDV outbreak in March, antibody titers (Table 3) and histopathological lesion scores (Table 4) were evaluated on both sides of the farm. The results indicated no significant differences in antibody titers against NDV, IBV, H5N1, or H9N2 between the front and back sides.

**Table 3:** The mean ± SE of the antibody titers against NDV, IBV, H5N1, and H9N2 and their statistical significance between the front and back sides of the laying farm during the outbreak in March.

Antibody titers	Front side	Back side	Sig.
NDV	10.72 ± 0.21	10.84 ± 0.22	0.708
IBV	3.42 ± 0.1	3.48 ± 0.1	0.735
H5N1	6.32 ± 0.11	6.3 ± 0.11	0.878
H9N2	6.46 ± 0.12	6.44 ± 0.15	0.885

**Table 4:** The mean ± SE of the lesion scores of the histopathologically examined organs and their statistical significance between the front and back sides of the laying farm during the outbreak that occurred in March.

Organs lesion score	Front side	Back side	Sig.
Trachea	4.02 ± 0.12	4.18 ± 0.13	0.328
Lung	2.58 ± 0.07	2.68 ± 0.07	0.303
Proventriculus	2.92 ± 0.09	2.96 ± 0.1	0.791

**Figure 6:**
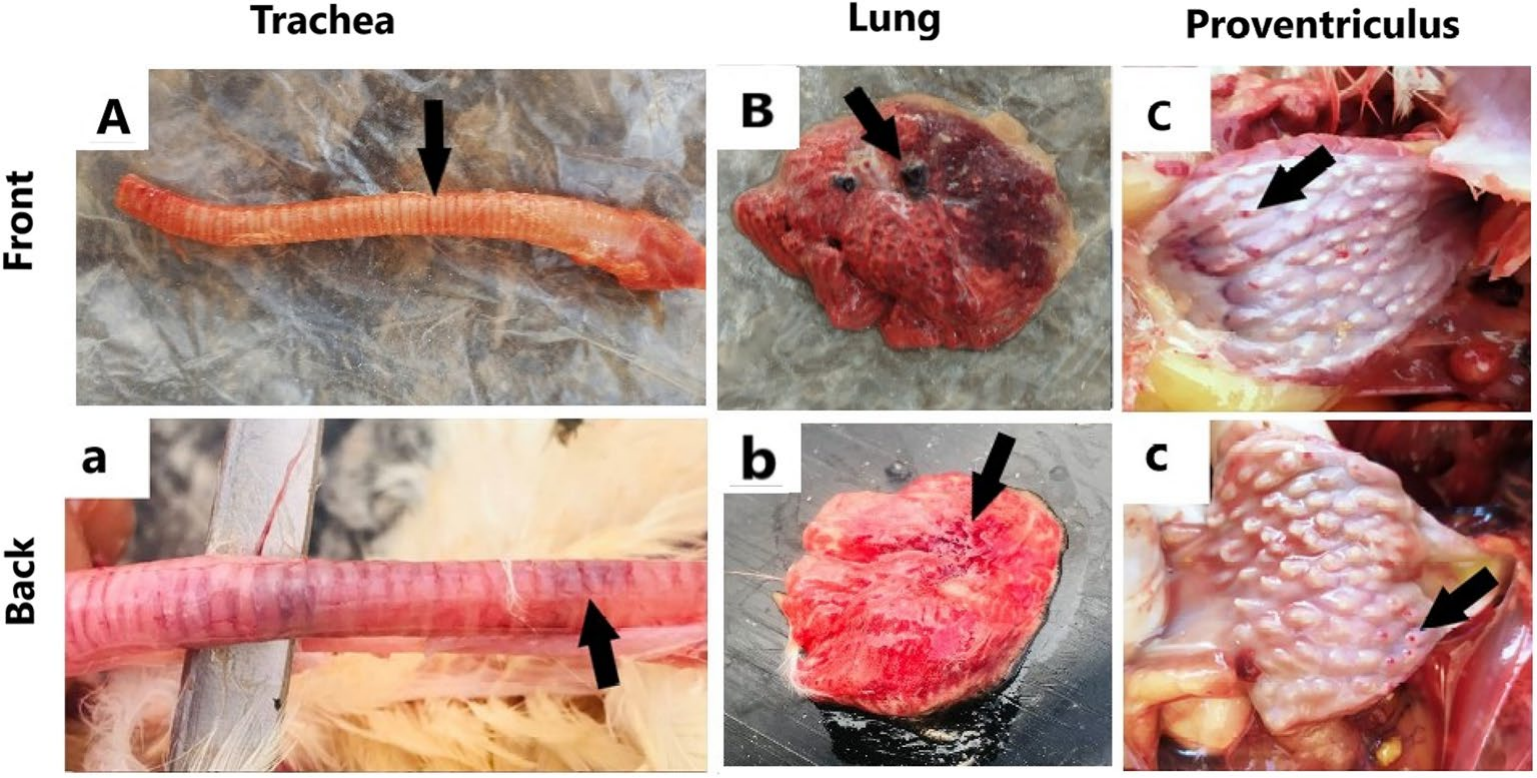
Gross pathological changes of the naturally NDV-infected laying hens. Lesions in the trachea, lung, and proventriculus were at the front (**A**–**C**) and back (**a**–**c**) side of the farm: (**A**) Congested tracheal mucosa (arrow), (**B**) Severe congestion and hyperemia of the lung tissue (arrow), (**C**) Hemorrhages (arrow) at the tip of the proventricular gland, (**a**) Congested tracheal mucosa (arrow), (**b**) Congestion and hyperemia of the lung tissue (arrow), (**c**) Hemorrhages (arrow) at the tip of the proventricular gland.

**Figure 7:**
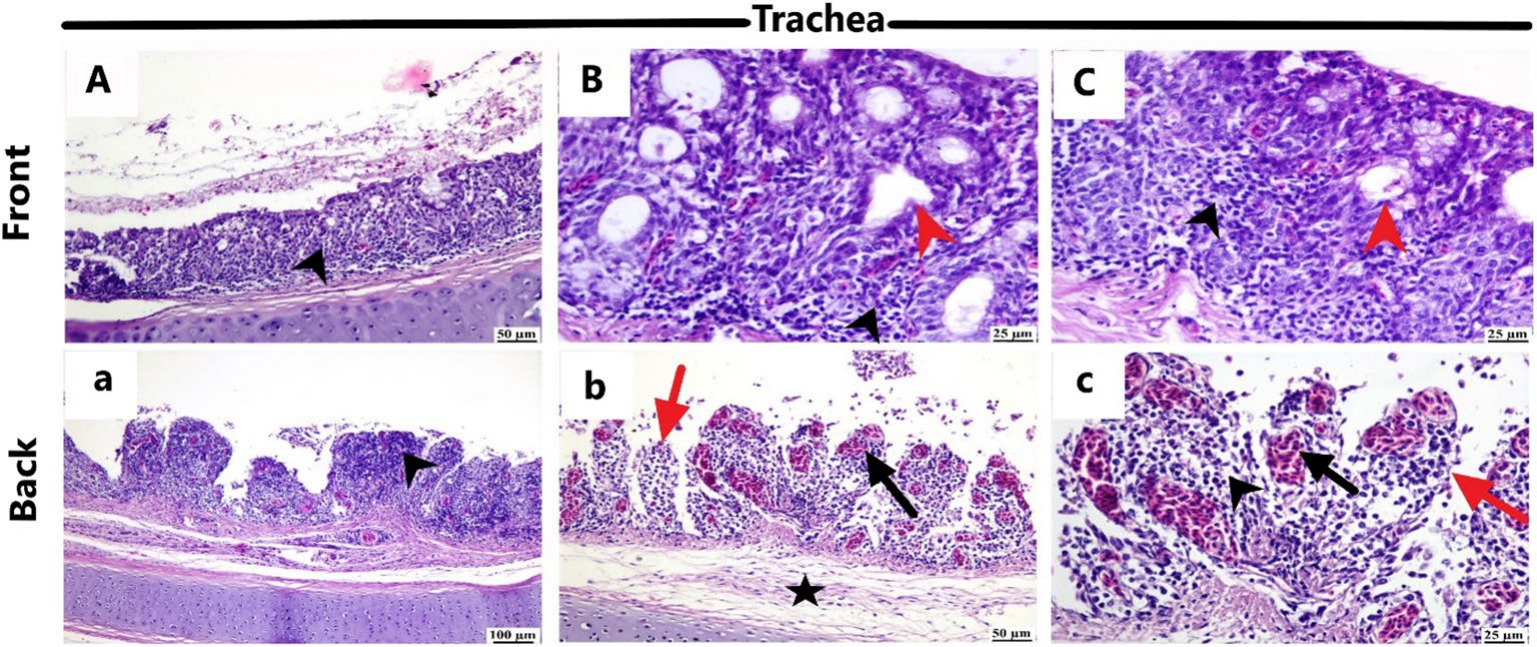
Histopathological changes in the trachea of the naturally NDV-infected laying hens at the front and backside of the farm. Front side: (**A**) Thickening of the tracheal mucosa with lymphoplasmacytic infiltration (black arrowhead); (**B**,**C**) mucosal ulceration with congested blood vessels, hyperplasia of the mucosal gland (red arrowhead), and lymphoplasmacytic infiltration (black arrowhead) (H&E). Back side: (**a**) severe desquamation of the tracheal epithelium with lymphoplasmacytic infiltration (black arrowhead); (**b**,**c**) Mucosal sloughing (red arrow), lamina propria and submucosa heavily infiltrated with inflammatory cells (black arrowhead) with extensive edema (asterisk) and congested blood vessels (black arrow) in lamina propria and submucosa (H&E).

**Figure 8:**
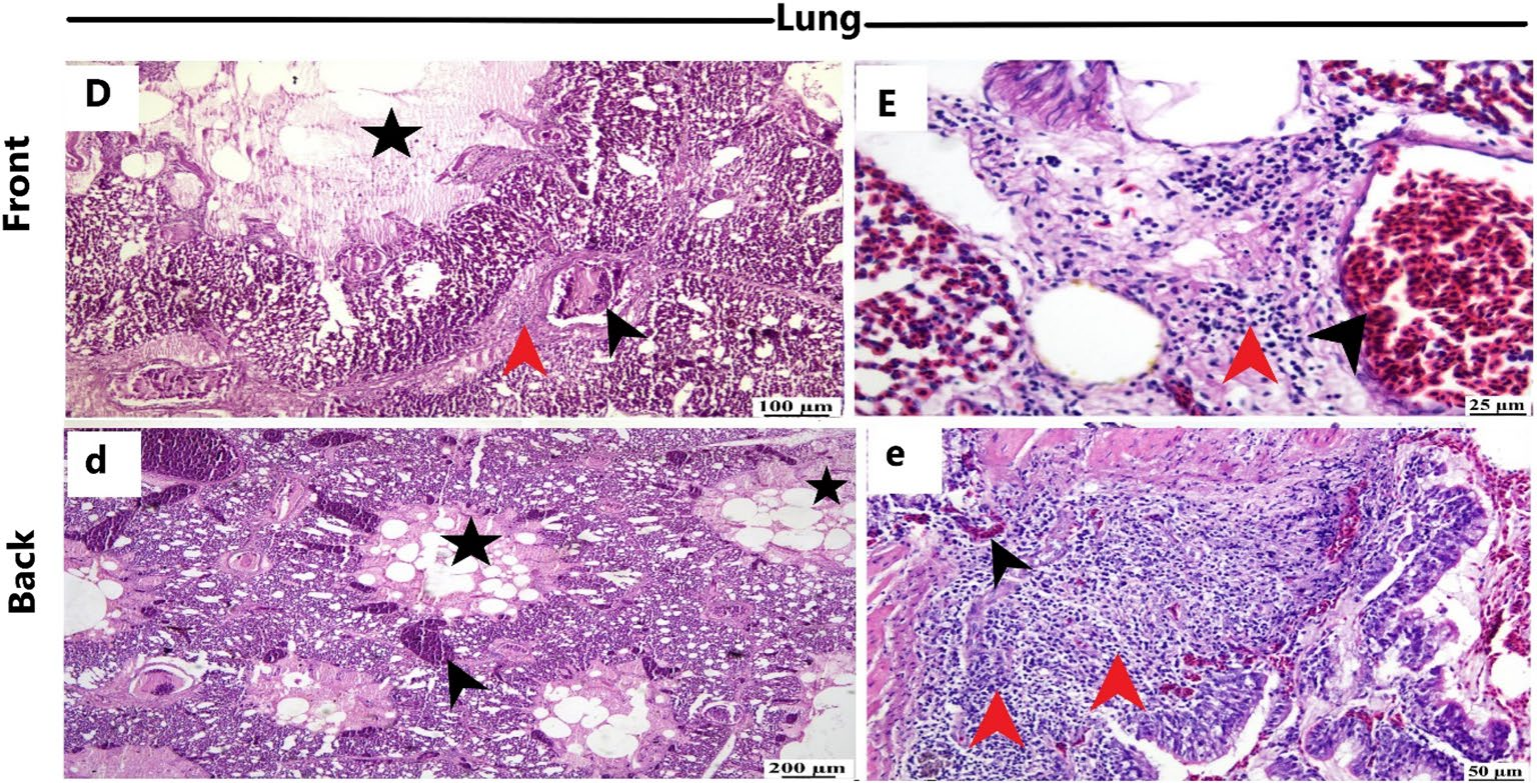
Histopathological changes in the lung of the naturally NDV-infected laying hens at the front and backside of the farm. Front side: (**D**,**E**) Parabronchus lumen filled with fibrin (asterisk), widening of the interstitial tissue with fibrin and inflammatory cells (red arrowhead), and congested blood vessels (black arrowhead) (H&E). Back side:(**d**) Diffuse fibrin exudation in the lumen of parabronchus (asterisk) and congested blood vessels (black arrowhead); (**e**) Thickening of the bronchiolar wall with inflammatory cells (red arrowhead) mainly heterophiles and lymphocytes extending to interstitial tissue (H&E).

**Figure 9:**
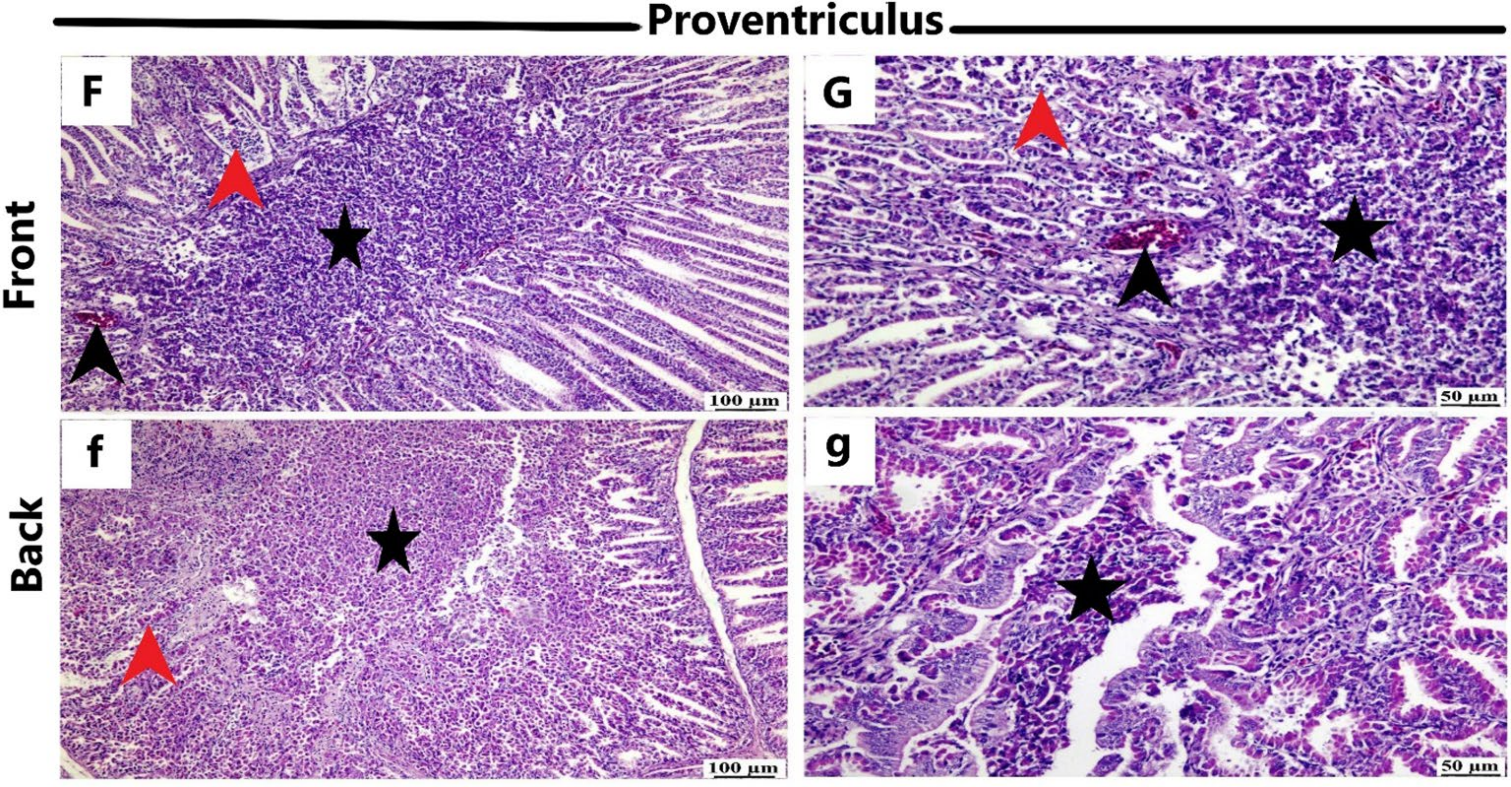
Histopathological changes in the proventriculus of the naturally NDV-infected laying hens at the front and back side of the farm. The front side (**F**,**G**) and backside (**f**,**g**) showed degeneration and necrosis of the acinar glands (red arrowhead), infiltration of the lumen with necrotic debris (asterisk) of mononuclear inflammatory cells and heterophile, and congested blood vessels (black arrowhead) (H&E).

Figure 6 presents the pathognomonic gross pathological changes in the trachea, lung, and proventriculus of the naturally NDV-infected laying hens. Histopathological examination (Figs. 7, 8, and 9) revealed lesions in the trachea, lung, and proventriculus. However, these lesions did not differ significantly between the two sides.

## Discussion

### Effect of microclimate on production indices

The study revealed significant variations in production indices, notably EPP and EW, between the front and back sides of the layer farm. These findings underscore the pivotal role of microclimate dynamics in influencing the laying performance of hens within a commercial poultry setup.

During the colder months, the front side consistently exhibited a marginally superior EPP. This phenomenon could be attributed to the presence of air inlets on the front side, optimizing the microclimate and reducing heat stress, which might occur through the accumulation of hot air at the air outlet and is known to adversely affect laying hens. This finding aligns with Sudjarwo et al., who highlighted the influence of microclimate conditions on EPP^5^. The mechanism behind this phenomenon is the cold air entering through the cooling pad, drawn in by the fan located at the end of the cage. Consequently, the area closer to the fan experiences higher temperatures than the region near the cooling pad. These temperature variations resulted in significant differences in the production performance of Hy-Line laying hens at different cage locations within the closed house, with the highest performance occurring in cages situated near the air inlet.

The slightly heavier eggs produced on the front side imply a potential advantage in productive performance. This finding aligns with the established understanding that hens subjected to milder environmental stressors tend to exhibit greater egg productivity. In February, the results show an increase in EW, followed by a decrease in March. This decline was followed by a gradual increase on both sides, ultimately reaching the highest EW in May. Additionally, during the winter, hen feed intake increased as the hens aged. Notably, EW is influenced by a significant interaction between the housing system and the age of hens, as reported by Vlckova et al.^28^. During March, the EW decreased in response to accidental NDV infection. However, through proper flock management, there was a gradual increase in EW over time.

Conversely, during warmer months, the back side demonstrated greater resilience in terms of EPP. This may be attributed to the need for additional cooling being reduced on the back side due to the airflow created by the front-side cooling system. Consequently, hens on the back side experienced less thermal stress, likely contributing to their robust laying performance. However, this robustness did not manifest in variations in EW, emphasizing the complex relationship between microclimate and EPP. As noted by Sharma et al., environmental activities have a significant impact on the growth performance and EPP of laying hens^29^. Climate change can have far-reaching effects on poultry, including affecting growth rates, appetite, feed utilization, EPP, and EW after Saeed et al.^30^.

Kocaman et al. reported that the ADFI and FCR in the summer and autumn months were lower than those in the winter and spring months, although the present study revealed a lower ADFI in autumn and spring than in winter^1^. This discrepancy may be attributed to the influence of climate change, as noted by Saeed et al.^30^, which can impact various factors, including growth rate, appetite, feed utilization, and EPP, in poultry. It is important to note that in commercial laying flocks, measurements of ADFI, BW, and EW cannot be conducted on an individual hen basis^12^. Achieving optimal and efficient production involves striking a balance between low ADFI and optimal EPP, as indicated by Sudjarwo et al.^5^.

The impact of AV (m/s) on EW is shown in Fig. 2, a positive association between AV (m/s) and EW on both the front and back sides suggested that well-regulated air circulation is conducive to superior egg productivity. The impact of AV (m/s) is significant, and it is essential to highlight that Ruzal et al. have stipulated that there is a maximum limit of 3 m/sec for this factor in hen houses^31^. Additionally, Purswell and Branton reported a positive correlation between AV and the EW mean, especially at a velocity of 0.76 m/s and a constant velocity of 1.52 m/s^32^.

Linear regression analysis revealed that AV was a significant predictor of front and back EW. It is imperative to note that the effect of AV on the EPP differed between the front and back sides. While AV had no significant impact on the EPP on the front side, it adversely affected the EPP on the back side. This dissimilarity may be attributed to the airflow pattern within the closed-house system, where excessive airflow could disrupt the comfort and laying routines of hens, ultimately affecting their productivity. This finding coincided with the findings of Sudjarwo et al.^5^. They noted significant differences in the effect of cage location, with the first cage location near the inlet in the closed-house system resulting in the highest egg mass. Furthermore, various studies, such as those by Bessei, Lara, and Rostagno, and Fernandes et al., have reported that the first cage location near the inlet is conducive to supporting good production performance of Hy-Line laying hens^33–35^.

Effective ventilation that manages heat and moisture can significantly impact EW, a critical factor in the marketability of eggs. However, linear regression analysis revealed that the indoor temperature, RH%, and THI had no effect and were not predictors of the indoor front or back EPP % or EW. In contrast, Ebeid et al. and Mack et al. reported that heat stress can lead to a significant reduction in EW by as much as -3.24% and a decrease in EPP^36,37^. Widjaya et al.^4^ noted that cage locations near cooling pads tend to have lower temperatures than those near exhaust fans. In contrast, the cage location near the inlet of the house maintains a cooler temperature. It is essential to note that the comfort zone for chickens typically falls within the temperature range of 18–25 °C, which effectively suppresses heat stress and allows the birds to maintain a normal body temperature of 41 °C. As emphasized by Sudjarwo et al., certain environmental factors, such as RH%, can have a substantial impact on EPP, contrary to the ideal conditions for laying hens suggested by Hafsa et al., which fall within the RH% range of 60–70%^5,38^. Kocaman et al.^1^ previously reported a strong negative correlation between the RH% and EPP, indicating that as the RH% in poultry houses increased, the EPP tended to decrease.

### Immune responses and histopathological changes

Despite the presence of several effective disease prevention protocols implemented on poultry farms, it remains a significant challenge to combat viruses that negatively impact EPP, posing a substantial hurdle for the global sustainability of the table egg industry. The obstacles faced in maintaining effective EPP can be attributed to a multitude of factors, which may encompass the infecting virus itself, the host organisms, and the management systems employed^39^.

The evaluation of humoral immune responses and histopathological changes during the March NDV outbreak offers crucial insights into hens' resilience to different microclimates. The results revealed no significant differences in antibody titers against NDV, infectious bronchitis virus (IBV), H5N1, or H9N2 between the front and back sides. This suggests that microclimate variations within the closed-house system did not significantly impact the hens’ immune responses to NDV or other common avian pathogens. This resilience in immune responses can be considered a promising aspect of poultry health management. Notably high levels of antibodies against NDV were detected on both the front and back sides serving as clear evidence of a robust humoral response generated in reaction to viral infection^40^.

Histopathological examination revealed consistent lesions in the trachea, lung, and proventriculus on both the front and back sides. The uniformity of these lesions implies that NDV infection affected hens similarly across microclimates within the closed-house system. The presence of characteristic NDV-associated histopathological changes, such as mucosal thickening, ulceration, and inflammatory cell infiltration in the trachea, as well as fibrin exudation and inflammatory cell infiltration in the lung, reinforces the diagnosis of NDV infection. The severe congestion and hyperemia of the lung, along with the degeneration and necrosis of acinar glands in the proventriculus, are consistent with established pathological responses to NDV infections in poultry. Microscopically, the tracheal sections from field-infected layer chickens exhibited congestion, hemorrhage, mononuclear inflammatory cell infiltration, and edema in nearly every tracheal sample. The lungs displayed signs of pneumonia, characterized by microscopic lesions featuring congestion, edema, and mononuclear inflammatory cell infiltration within the alveoli and their walls. Furthermore, in the proventriculus, epithelial mucosal cells, submucosal inflammatory cells, and glandular epithelial cells all exhibited immunopositivity for NDV^13^.

## Conclusions

Air velocity is the best microclimatic player inside the closed houses of laying hens and controls other microclimatic parameters. Effective ventilation, which affects the indoor ambient temperature and RH%, can significantly impact egg weight. Without the air velocity, the indoor temperature, RH%, and THI had no effect and did not act as predictors of the percentage of egg production or egg weight on the front or back side of the farm. A system for ongoing monitoring of microclimate conditions, production indices, and immune responses in laying hens must be established.

## Data Availability

The datasets used and/or analyzed during the current study are available from the corresponding author upon reasonable request.
